# A pilot Internet "Value of Health" Panel: recruitment, participation and compliance

**DOI:** 10.1186/1477-7525-4-90

**Published:** 2006-11-27

**Authors:** Ken Stein, Matthew Dyer, Tania Crabb, Ruairidh Milne, Alison Round, Julie Ratcliffe, John Brazier

**Affiliations:** 1 Peninsula Technology Assessment Group, Peninsula Medical School, University of Exeter, Noy Scott House, Barrack Road, Exeter, EX2 5DW, UK; 2 National Coordinating Centre for Health Technology Assessment, University of Southampton, Boldrewood, Bassett Crescent East, Southampton, SO16 7PX, UK; 3 Sheffield Health Economics Group, School of Health and Related Research (ScHARR), University of Sheffield, 30 Regent Court, Sheffield, S1 4DA, UK

## Abstract

**Objectives:**

To pilot using a panel of members of the public to provide preference data via the Internet

**Methods:**

A stratified random sample of members of the general public was recruited and familiarised with the standard gamble procedure using an Internet based tool. Health states were perdiodically presented in "sets" corresponding to different conditions, during the study. The following were described: Recruitment (proportion of people approached who were trained); Participation (a) the proportion of people trained who provided any preferences and (b) the proportion of panel members who contributed to each "set" of values; and Compliance (the proportion, per participant, of preference tasks which were completed). The influence of covariates on these outcomes was investigated using univariate and multivariate analyses.

**Results:**

A panel of 112 people was recruited. 23% of those approached (n = 5,320) responded to the invitation, and 24% of respondents (n = 1,215) were willing to participate (net = 5.5%). However, eventual recruitment rates, following training, were low (2.1% of those approached). Recruitment from areas of high socioeconomic deprivation and among ethnic minority communities was low. Eighteen sets of health state descriptions were considered over 14 months. 74% of panel members carried out at least one valuation task. People from areas of higher socioeconomic deprivation and unmarried people were less likely to participate. An average of 41% of panel members expressed preferences on each set of descriptions. Compliance ranged from 3% to 100%.

**Conclusion:**

It is feasible to establish a panel of members of the general public to express preferences on a wide range of health state descriptions using the Internet, although differential recruitment and attrition are important challenges. Particular attention to recruitment and retention in areas of high socioeconomic deprivation and among ethnic minority communities is necessary. Nevertheless, the panel approach to preference measurement using the Internet offers the potential to provide specific utility data in a responsive manner for use in economic evaluations and to address some of the outstanding methodological uncertainties in this field.

## Background

Although concerns have been expressed about the use of cost utility analyses (CUA)[1,2], the number of such analyses has increased in the past ten years[3]. Guidelines in the UK and Canada, and those proposed by the Washington Panel on cost effectiveness in the USA, promote CUA where the purpose of the analysis is informing public resource allocation [4-6] The UK's National Institute for Health and Clinical Excellence (NICE) has made cost utility an explicit aspect of policy making[6]. The UK and Washington Panel reference cases suggest that the perspective for the valuation of benefits in CUA should be that of the general public[5,6]. The arguments around adopting this perspective are beyond the scope of this article, but are described elsewhere[5,7-14,14]

A wide range of approaches has been taken to obtain utility data for economic evaluations[15] Although the widespread use of standard measures such as the EQ5D and SF6D[16] may address some of this inconsistency, this approach will not be appropriate in all situations and there remains a case for developing alternative methods for obtaining health state-specific utility data. We have piloted one approach, using the Internet to obtain preferences on written health state descriptions from a "standing panel" of members of the public.

Computer-based preference elicitation tools have been available for more than 15 years [17-23] with later use of the Internet [24-28]. Many preference elicitation tools, and studies employing them, are concerned with the psychology of preference elicitation[29,30] and are therefore less concerned with selection bias than Internet-based epidemiological[31,32], behavioural[33,34] or therapeutic studies[35,36]. While Internet based research faces many of the same challenges encountered in more traditional approaches, additional concerns are legitimate, in particular: sampling and sampling representativeness, competition for the attention of respondents, and barriers to participation related to literacy or disability[37]. Reported experience varies, with some studies reporting very disappointing results for recruitment and retention[38], and others showing rates which are comparable to traditional methods[39,40]. However, despite possible exceptions[31], it seems reasonably consistent that research participants in Internet-based studies are likely to be different from those recruited by other means [41-44]. Whether these differences matter in the context of preference elicitation studies remains uncertain.

In this paper we describe recruitment and participation in the pilot panel study and discuss the potential for extension of this approach to fulfil the need for eliciting utilities from the general public for research purposes and to support the need for these values to inform allocation policy decisions.

## Methods

### Recruitment and training

We recruited panel members from a convenience sample of four UK cities: Exeter, Sheffield, Glasgow and Aberdeen. A random sample was chosen from the electoral rolls for these cities in January 2004, stratified for socio-economic status using tertiles of the Index for Material Deprivation (IMD2000)[45]. We assumed a 15–20% response rate to the invitation to attend panel training based on the authors' previous experience with preference elicitation studies using face to face interviews and aimed for an arbitrary target sample size for the panel of 100.

Participants were invited by letter to express interest in joining the panel, accompanied with a short questionnaire seeking reasons for non-participation. Positive respondents were then invited to a three hour training session in each of the cities involved. Panel members were recruited and trained in two phases during summer and autumn 2004, involving eight training sessions.

Training sessions covered the following areas as background: research and policy making; role of modelling in estimating cost effectiveness; limitations of existing methods for utility assessment. Participants were familiarised with the standard gamble, using formats appropriate to whether the health states were considered better or worse than death, with one-to-one support from facilitators.

Health state descriptions were placed on the website for at least three weeks. Descriptions were posted on the website in sets containing different health states within the same condition (e.g. levels of severity or treatment side effects). States within a set were presented in random order. Sets included health states depicting the following diseases: congestive heart failure; eczema; hip osteoarthritis; Crohn's disease; colorectal cancer; depression; glioma; prostate cancer; insomnia; ovarian cancer; opiate abuse; and chronic obstructive pulmonary disease. Descriptions were developed using reports of quality of life using patient based disease-specific outcome measures and clinical expert opinion and presented in bullet point rather than narrative format[46].

We encouraged participants by email to provide preference values in this period and issued email reminders. Panel members who valued at least one description within the three week period were entered into a lottery for £50 Internet gift vouchers, held after each set of descriptions were taken off the Internet site. A regular newsletter was sent to participants reporting participation, results, website developments and other news regarding the project.

### Preference elicitation

Panel members were asked to imagine themselves in the described health state, for at least twenty years, or, if they felt their life expectancy was likely to be less than that, for the rest of their life[47] The standard gamble method was used, based on the axiomatic advantage that it reflects choices made under conditions of uncertainty[48] This was carried out using bottom-up "titration", in which respondents work through choices with increasing probability of good outcome in the gamble option. We used this approach rather than an iterative approach where responses "ping-pong" between options with high and low probabilities of worst outcome in the gamble[49] in order to overcome reported difficulties with completion of the iterative approach[46].

### Internet site development

The website was created in 2004 and piloted by the project team and panel members from the first phase of recruitment. It includes the standard gamble interface, information on the project, and a bulletin board for sharing questions and information on the project.

**Figure 1:**
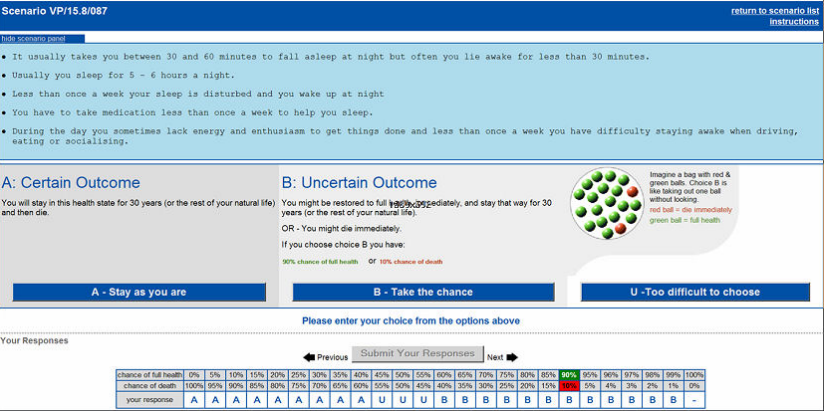
Standard gamble interface.

The standard gamble interface (Figure 1) has several features of interest:

- It is not possible for participants to enter responses which are fundamentally illogical e.g. preferring the gamble at a given probability of restoration of full health, but then preferring the health state of interest when this probability increases. (Do you jump right into a gamble or start with a question asking preference between perfect health and the state of interest?)

- Participants who indicate that they would take the gamble where the probability of death is 1.0 must confirm that they consider the health state description worse than being dead. They are then automatically taken to an interface which presents the options appropriately for the elicitation of negative utility values.

- As the probabilities in the risky choice change, they are represented graphically as a bag of different coloured balls, each representing the potential outcomes of full health and death.

- Participants had three possible respondes to each choices in the standard gamble: choose to remain in the described health state; choose the risky option (with varying chance of death or full health); or "uncertain". Illogical chains of response (e.g. "remain in health state", followed by "uncertain", followed by "remain in health state") were not permitted and participants were required to repeat the choice which resulted in the illogical response. Choices at all levels of risk had to be completed before the response was accepted.

- The increments for changing probability in the gamble are set at 1% between probabilities of full health of 0.95 and 1.0 in the gamble option and 5% otherwise.

Responses were downloaded into a database with automatic calculation of respondent's utility for each health state description.

### Analyses

Recruitment was described and the demographic characteristics of the pilot panel compared to data from the UK National Census carried out in 2001.

Completion of preference elicitation tasks was described in three ways. First, *participation by panel member*, defined as the proportion of panel members who carried out at least one valuation task during the study period. Second, for each set of health state descriptions, the proportion of panel members who responded was calculated – *participation by health state description set*. Third, for each panel member who carried out at least one valuation task (participant), we calculated *compliance*, defined as the proportion of health states valued by each participant.

Potential determinants of participation by panel member and compliance were explored through univariate and multivariate analyses using SPSS for windows version 11. Age, marital status, occupation and ethnicity were collected from panel members at recruitment. Socioeconomic status was attributed according to place of residence, using the Scottish Index of Material Deprivation (SIMD) for Aberdeen and Glasgow[50], calculated at postcode sector level and the 2004 version of the Index of Material Deprivation for Exeter and Sheffield at Lower Super Output Area (LSOA) level[51]. LSOAs contain populations of 1000–1500 people. For the purposes of the analysis, SIMD and IMD were treated as a single scale. Other variables considered were city of residence, nationality (Scottish or English) and training session.

## Results

### Recruitment and retention

Recruitment was carried out in two rounds. Initially, people in Exeter, Sheffield and Aberdeen were recruited and trained. It became clear that the target panel size would not be met from this sample and a further round of recruitment took place in Exeter and Glasgow to increase panel size. Overall, recruitment and training took about seven months. The panel carried out valuation tasks from August 2004 through March 2006, and we met our membership (n = 112) goal in November 2004. In Autumn 2004, therefore, we were recruiting new panel members while existing members were participating in valuation tasks.

Overall, 5,320 people were contacted through the electoral roll. Only 1215 (23%) of those approached responded to the initial invitation letter. Of this group, 286 (23.6%) expressed willingness to participate in the project and 112 (39% of those who agreed) attended a training session. Only people who attended a training session were considered part of the panel. Thus, the net final recruitment was 2.1% of those initially approached.

Residents from Exeter were more willing to participate (see Table 1: χ^2 ^= 41.18, P < 0.001) and were more willing to give reasons for declining (see Table 2: χ^2 ^= 12.86, P < 0.001) compared to residents from the other cities. Lack of Internet access was more frequently reported among respondents in cities other than Exeter (χ^2 ^= 62.0, P < 0.001). Lack of time and Internet access were the most common reasons given for declining the initial invitation to participate. Other reasons included illness or disability, impending travel and not reaching the intended recipient because of incorrect address details or their decease.

**Table 1:** Recruitment by City

	**Exeter**	**Sheffield**	**Glasgow**	**Aberdeen**	**All sites**
	**N (%)**				

Sent invitation letter	1892	1892	1000	536	5320
Positive response to invitation letter	151 (8.0%)	84 (4.4%)	29 (2.9%)	22 (4.1%)	286 (5.4%)
Attended training i.e. joined panel	72 (3.8%)	22 (1.2%)	11 (1.1%)	7 (1.3%)	112 (2.1%)
Gave reasons for declining initial invitation	263 (13.9%)	210 (11.1%)	98 (9.8%)	55 (10.3%)	626 (11.8%)

**Table 2:** Reasons for declining initial invitation

	Exeter	Sheffield	Glasgow	Aberdeen	All cities
Reason for not participating	N (%)				

Don't understand the project	7 (3)	8 (4)	0	4 (7)	19 (3)
Not interested	7 (3)	7 (3)	4 (4)	3 (5)	21 (3)
Don't have time	101 (38)	70 (33)	30 (31)	16 (29)	217 (35)
No access to the Internet	49 (19)	100 (48)	51 (52)	28 (51)	228 (36)
Other	99 (38)	25 (12)	13 (13)	4 (7)	141 (23)
Total	263	210	98	55	626

### Panel member characteristics

**Figure 2:**
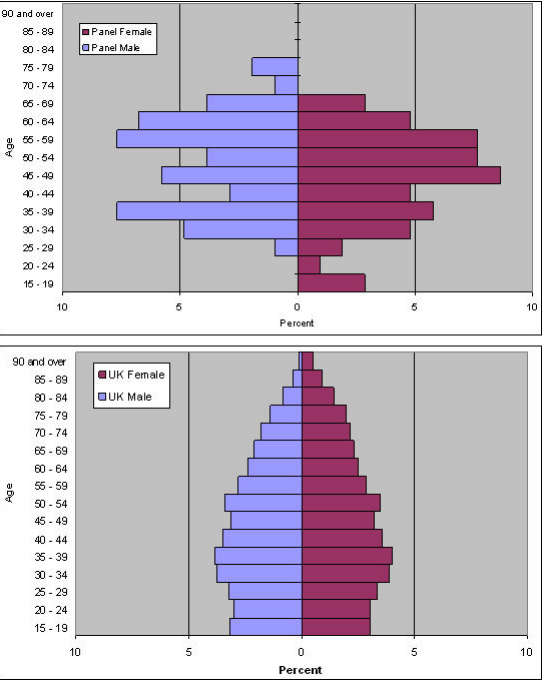
Value of Health Panel age structure vs UK population.

The age range of panel members was 18 to 79 years with mean 48 years. The panel included a higher proportion of people in middle age than the UK population as a whole, and fewer younger and older people (see Figure 2).

Table 3 shows the demographic characteristics of the panel members. There were more women (51.8%) than men (48.8%) (P = NS). Men were, on average, slightly older than women, though the difference was not significant. The panel had a higher proportion of married and retired people with correspondingly lower proportions of unmarried people and those in employment than the national population. However, only the proportions of married and single people and those from ethnic minorities were differed significantly from national data

**Table 3:** Panel member personal characteristics

	Panel Characteristics							
	Males		Females		Total			
	Mean	SD	Mean	SD	Mean	SD	National (England and Scotland)	Difference between Panel (total) and National^a^
**Age**	50.5	13.4	46.0	13.0	48.2	13.3		
	N	%	N	%	N	%	%	% (P)

**Employment**								
Student	2	3.7	5	8.6	7	6.3	8.2	-0.7 (NS)
Full-time	25	46.3	22	37.9	47	42.0	55.7	-5.2 (NS)
Part-time	2	3.7	13	22.4	15	13.4	16.0	0.2 (NS)
Unemployed	0	0	3	5.2	3	2.7	4.0	-0.8 (NS)
Retired	14	25.9	7	12.1	21	18.8	16.0	6.5 (NS)
Other	5	9.3	5	8.6	10	8.9		
Unknown	6	11.1	3	5.2	9	8.0		
**Marital Status**								
Married	33	61.1	37	63.8	71	68.3	40.6	27.7 (<0.05)
Single	11	20.4	10	17.2	21	18.8	44.5	-24.1 (<0.05)
Divorced	3	5.6	5	8.6	8	7.1	6.3	1.4 (NS)
Separated	1	1.9	1	1.7	2	1.8	2.0	-0.002 (NS)
Widow	1	1.9	1	1.7	2	1.8	6.6	-4.8 (NS)
Unknown	5	9.3	4	6.9	9	8.0		
**Ethnicity**								
White	44	81.5	42	72.4	86	76.8	91.4	-14.6 (<0.05)
Non-white	0	0.0	1	1.7	1	0.9	8.6	-7.7 (<0.05)
Unknown	10	18.5	15	25.9	25	22.3		

^a ^Proportions for Panel data are calculated excluding categories not reported in National data to ensure comparability

Table 4 shows the proportion of panel members from each city whose area of residence fell into tertiles of IMD or SIMD scores ranked at a national level for Scotland or England. The distribution is significant (χ = 16.8, P < 0.025). If the panel reflected the national distribution of socioeconomic status as measured by the IMD/SIMD, the samples from each city would contain 33% of people in each national tertile. People from areas of high deprivation are under-represented in the panel, particularly in Exeter and Sheffield. The numbers of people recruited from Scotland were low, making this comparison imprecise.

**Table 4:** Panel compared to national distribution of socioeconomic status

	N (%) Panel members whose residence falls into national tertiles of IMD* or SIMD*		
**City**	**High**	**Medium**	**Low**

Exeter	12 (16.2)	26 (35.1)	34 (45.9)
Sheffield	4 (18.2)	7 (31.8)	11 (50.0)
Glasgow	7 (63.6)	3 (27.3)	1 (9.1)
Aberdeen	2 (28.6)	0	5 (71.4)
Total	25 (22.3)	36 (32.1)	51 (45.5)

High = most socioeconomic deprivation

Low = least socioeconomic deprivation

### Participation and compliance

During the first year of the project (October 2004–5), 25 members (22%) of the panel formally withdrew. Most of these panellists had completed some valuations before withdrawal. Having insufficient time, moving house, losing Internet access and personal or family illness were the main reasons cited. There was no statistical association between age, sex or socioeconomic status and this explicit withdrawal from the project.

Overall, 83 panel members (74.1%) participated in the project i.e. carried out at least one valuation. In almost all cases (94.5%), panellists who completed one health state in a set of health states, went on to complete the entire set. Most valuation tasks were carried out in one sitting: in only 13 (2.3%) were responses from a set received on more than one day. In these cases, respondents carried out valuations in no more than two sittings separated by 1 to 28 days (mean 6.9 days, median 6 days).

**Figure 3:**
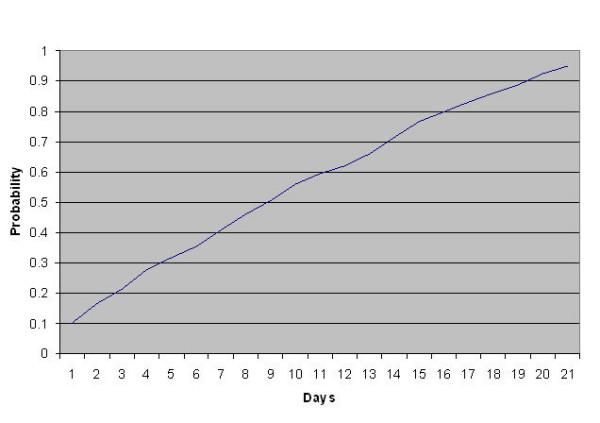
Probability of participation within 21 days of a set of health state descriptions being posted.

Panel members were asked to complete the valuation tasks within an arbitrary three week period, although in some cases descriptions were posted for longer. Figure 3 shows the cumulative probability of obtaining a set of values within 21 days. Where respondents carried out valuation on more than one day, the date of completion (i.e. the second date) was used in this calculation.

**Figure 4:**
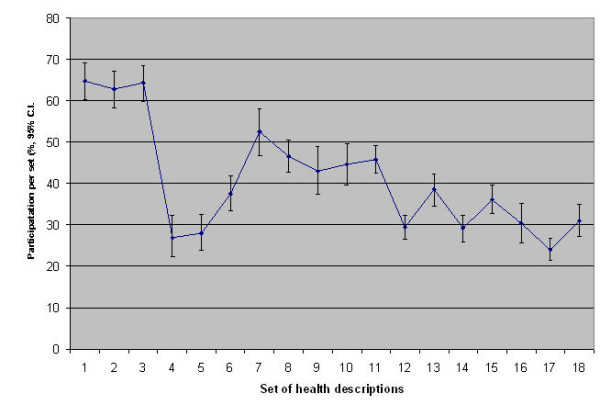
Participation over time.

Taking variations in panel membership into account, overall average participation by health state description set was 41% (range 24%–65%). This is the proportion of available panel members who completed each set of health state descriptions (see Figure 4). The drop in participation around presentation of the third set of decriptions results from a combination of (a) increased panel membership following the second round of recruitment and (b) initial access problems experienced by new panel members, mostly related to incorrect email addresses and incorrect or forgotten logins and passwords. Resolution of these problems resulted in an increase in participation, although this was followed by a gradual decline.

Univariate analysis showed no significant association with participation and age, sex, nationality, city, retirement status or training session. Data on ethnicity were incomplete and excluded from further analysis.

People with lower socioeconomic status were less likely to participate (t test, t = 3.713, P = 0.013) and those who were married were more likely to participate; 86% of married people participated versus 52.5% of unmarried people (χ^2 ^= 13.90, P < 0.001).

Logistic regression confirmed the independent effects of socioeconomic status and marital status on participation. The odds ratio (95% confidence interval) for marital status was 0.57 (0.36 to 0.91), although odds ratios for specific categories were not significant. This analysis is therefore akin to a χ^2 ^test for trend. In the same model, the odds ratio for participation according to IMD score was 0.94 (0.91 to 0.98) i.e. the odds of participation fell slightly as IMD (socioeconomic deprivation) increases. Pseudo-R^2 ^for the model was low, at 0.12.

**Figure 5:**
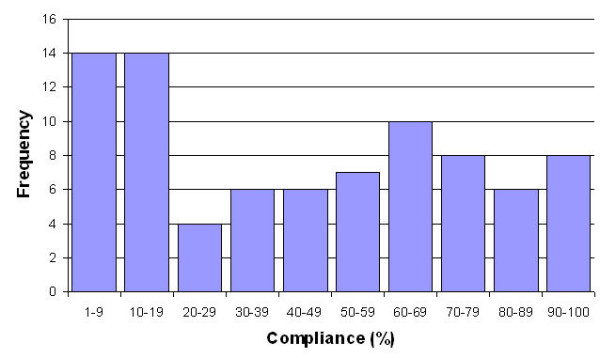
Distribution of compliance.

Compliance, defined as the proportion of health state valuations provided by each member as a percentage of the total for which they were eligible to complete, ranged from 3–100% (see Figure 5). A quarter of the panel carried out less than 20% of the elicitation tasks. There was no association between compliance and age (Spearman correlation, P = 0.92); sex (t test, P = 0.422); nationality (ANOVA, P = 0.23); city (ANOVA, P = 0.631); marital status (t test, P = 0.568); occupation (ANOVA, P = 0.19) or IMD/SIMD score (Spearman correlation, P = 0.40).

## Discussion

This is the first attempt, of which we are aware, to collect new utility data repeatedly from members of the public for the specific purpose of informing ongoing cost utility analyses. Although we have demonstrated basic feasibility, in so far as the panel was established as planned and utility data obtained within the required period, recruitment was very low and retention limited. This was, in part, driven by the need for attendence at a training session. Initial positive response to the invitation to participate was similar to that shown in studies previously carried out by one of the authors (JB) aiming to recruit for a single episode of health state valuation using face to face interviews.

Across health state description sets, participation was around 40%, giving a sample size range for each health state description of 28 to 62. Participation by health state description set declined during the study period, demonstrating the need for ongoing recruitment and training. However, around 30% of the panel continued to participate at one year, and appeared to stabilise, consistent with other accounts of Internet research[52]. It is perhaps not surprising that recruitment and retention were limited given the burden placed on respondents: to attend face to face training and respond to 18 sets of preference measurement with limited rewards (a small cash lottery).

Reips identified 25 advantages and disadvantages (and proposed solutions) of the Internet for psychological experiments[52]. Our study avoided the problem of multiple submissions by requiring logging into the standard gamble and checking the timing of submissions, and the potential for misunderstanding through lack of interaction was addressed by initial training sessions. However, drop out remained high despite the use of financial incentives, reminders, some personalisation and limited feedback. Feedback from the panel suggested that more detailed and personalised feedback on their utility data and the purposes to which they were put, and a certain payment rather than a lottery may have improved compliance.

The three week period chosen for valuation tasks was arbitrary but appears appropriate. The probability of completion by that time was very high, even where health state descriptions were available on the website for longer. This issue has not been addressed in previous studies. The shape of the curve for completion was surprising. We expected there would be an initial surge of responses after descriptions were posted which would quickly tail off, with smaller responses following reminders. Reminders were sent at varying points while each health care description set was posted on the Internet and this may account for the overall pattern shown i.e. that panellists carried out the valuation tasks fairly evenly throughout the three week period.

The demographic make up of this pilot panel does not reflect Scotland and England as a whole. This was not unexpected: one of the purposes of the pilot study was to understand better the determinants of recruitment, participation and compliance so as to inform the establishment of a larger, more representative panel. Representation of people from more deprived areas, and from ethnic minority groups, was particularly low, demonstrating the challenge for engagement which is shown in other types of study[53] This was despite stratification by socioeconomic status.

In addition to the low initial recruitment from areas of higher socioeconomic deprivation, lack of participation amongst people recruited to the panel was also associated with lower socioeconomic status. The association between marital status and participation is not explained by covariance with the other limited independent variables. Surprisingly, compliance was not associated with socioeconomic status, suggesting either that the number of participants was insufficiently large to demonstrate an effect, or that the principal impact of socioeconomic status is on participation. Lack of adequate access to the Internet or lack of effectiveness in training sessions would be consistent with the latter hypothesis. The association between participation and marital status was not shown for compliance, which showed no association with any of the other covariates measured.

The importance of the panel's lack of representativeness depends on the influence of demographic factors on utilities for hypothetical conditions, which is an area of limited previous study. Age [54-56], sex[54,56], marital status[54], nationality[57], educational level[58] and ethnicity[59] have been demonstrated as being significant predictors of utility. Experience of illness appears to be a particularly important determinant of variation in preferences for hypothetical states [60-62].

The underlying reasons for variation in utilities for hypothetical states is not clear but may relate to risk attitude[63], whose distribution is very unclear in the general population, or numeracy[64].

The extent to which the panel's utilities represent what would be obtained from a demographically representative panel is therefore unclear and may not, relative to other concerns, be of paramount importance. Firstly, most research to date has focussed on the effect of demographic factors on the absolute utilities for health states, rather than on the impact of these factors on the effect of moving between states. It is not clear, therefore, with the possible exception of current illness[62], whether demographic imbalance would result in different estimates of incremental effectiveness between health technologies competing for scarce health care resources. Secondly, variation in utilities arising from methodological factors (e.g. choice of rating task, perspective of rater) appear to be more influential. This suggests that, while analysts might be cautious about using utilities from a source which is not demographically balanced, they should be more averse to combining utilities from sources which use different methods in the same evaluation.

The use of computer-based preference elicitation is not new[17]. Sumner *et al *developed the Utiter programme in 1991[18]. This was followed by U-Maker[19], Gambler[20], iMPACT[21,22] and, more recently, ProSPEQT[23]. In addition, "one-off" computer based utility assessment has been used in a wide range of studies [65-67] and as a teaching tool[68]. Computer based utility measurement has potential advantages over interviewer-based methods: lower cost once software has been developed; elimination of interviewer variation; avoidance of transcription errors in data entry; potential to address logical errors automatically[22]; and increased flexibility over the time required to complete the task. Acceptability among members of the general public is reasonable, although the standard gamble has been rated as less acceptable than visual analogue scaling or time trade off in one study[69]

The use of the Internet is a logical extension to the development of computer-based utility measurement tools. The most technically sophisticated approach is iMPACT3, developed by Lenert and colleagues. This uses an object orientated approach to facilitate the depiction of health states using written descriptions or multi-media presentations[24] and includes automatic error correction[70] Ubel and colleagues have also developed a series of Internet-based tools, including the person trade off[71] for use in a range of experiments [25-28].

Lenert[72] suggests web based preference elicitation may reduce interviewer bias, although we are not aware of studies which have addressed this using the standard gamble. However, Damschroder *et al*[71] have compared computer based preference measurement using PTO to face to face interview and found no significant differences in: values obtained; occurrence of non-trading; or measures of logical consistency between the two modes.

Although the Value of Health Panel project shares many of the features of other Internet based preference measurement systems, it is unique in having recruited and maintained a group of members of the public who have expressed preferences on a wide range of health state descriptions. Recruitment was, however, not Internet-based. There are no published accounts of recruitment to preference studies using the Internet, although Ubel *et al *have reported obtaining a large representative sample of US citizens for one study[26] Validation of the data obtained from such panels remains important, and logical consistency and procedural invariance are methods which may be applied[73]. Although some work has been carried out in this project[74], the area remains relatively under-studied in general.

The establishment of Internet panels for market research has increased dramatically in the past five years. Harris Interactive, advertise a global panel of 1 million members, with 600,000 in the USA[75]. In the UK, YouGov has recruited a panel of 89,000 people through Internet advertising and floated on the Stock Exchange in 2005 [76]. However, Internet penetration in the UK is only around 52% and people who are likely to join Internet panels are more likely to be politically interested and knowledgeable than those less likely to participate[77].

Nevertheless, it seems likely that the upward trend in Internet access will continue, as will access to broadband technology. This presents important opportunities for preference measurement and research with, potentially, advantages over one-to-one interviews. For example, large numbers of people can be involved; alternatives to written descriptions can be used; costs are likely to be less than one to one interviews; automatic checks for illogical responses can be integrated; and various approaches to representing risk (or time) in preference measurement can be explored. In short, the potential for using the Internet in this field, to improve the application of cost utility analyses and address some of the important methodological challenges that exist in preference measurement, is only beginning to be exploited.

## Authors' contributions

KS, RM, JB, JR and AR conceived the study and participated in its design.

KS coordinated the studied, participated in statistical analyses and drafted the manuscript.

MD and TC participated in recruitment, health state development and statistical analyses.

All authors read and approved the final manuscript.

## Funding

NHS R&D Programme; National Institute for Health and Clinical Excellence (NICE); NHS Quality Improvement Scotland (NHSQIS).
